# An efficient planar accordion-shaped micromixer: from biochemical mixing to biological application

**DOI:** 10.1038/srep17876

**Published:** 2015-12-14

**Authors:** Armando Cosentino, Hojjat Madadi, Paola Vergara, Raffaele Vecchione, Filippo Causa, Paolo Antonio Netti

**Affiliations:** 1 Center for Advanced Biomaterials for Healthcare@CRIB, Istituto Italiano di Tecnologia (IIT), Largo Barsanti e Matteucci 53, 80125 Naples, Italy; 2 Interdisciplinary Research Centre on Biomaterials (CRIB), University “Federico II”, Piazzale Tecchio 80, 80125 Naples, Italy; 3 Dipartimento di Ingegneria Chimica, dei Materiali e della Produzione Industriale (DICMAPI), University “Federico II”, Piazzale Tecchio 80, 80125 Naples, Italy

## Abstract

Micromixers are the key component that allow lab-on-a-chip and micro total analysis systems to reach the correct level of mixing for any given process. This paper proposes a novel, simple, passive micromixer design characterized by a planar accordion-shape geometry. The geometrical characteristics of the presented design were analyzed numerically in the range of 0.01 < Re < 100 based on the micromixer performance. The performance of the most efficient design was experimentally investigated by means of fluorescence microscopy for a range of low diffusion coefficients, 10^−12^ < D < 10^−11^ m^2^/s. The micromixer structure was fabricated in a simple single-step process using maskless lithography and soft lithography. The experimental results showed a very good agreement with the predicted numerical results. This micromixer design including a single serpentine unit (1-SERP) displayed an efficiency higher than 90% (mixing length = 6.4 mm) creating a pressure drop of about 500 Pa at Re = 0.1 and 60 kPa at Re = 10. A mixing efficiency of almost 100% was readily reached when three serpentine units were included (3-SERP). Finally, the potential diagnostic value of the presented microdevice was validated experimentally for Red Blood Cell (RBC) lysis.

Micromixers are a key component in several Lab On a Chip (LOC^1^) and Micrototal Analysis Systems (*μ*TAS), which are increasingly being used in chemical, biological and biomedical applications^2^,^3^,^4^,^5^,^6^,^7^. Mixing efficiency and rapid mixing play a major role in the characterization and performance of microfluidic systems; researchers have been making numerous attempts to develop a versatile and efficient mixing mechanism^8^,^9^,^10^. For microfluidic devices, the Reynolds number is small (≪100), *i.e.* the viscous effects dominate inertial effects, a completely laminar flow occurs where fluid streamlines are parallel to each other and convective mass transfer occurs only in the direction of the fluid flow. Consequently, the microfluidic mixing mechanism is dominated by diffusion, which is based on the concentration gradient, a process that leads to the increment of the time and length of the mixing procedure. Presently, micromixer designs and technologies are classified into active and passive micromixers^9^,^10^. Active micromixers use an external source of energy^11^,^12^,^13^,^14^,^15^,^16^, while passive micromixers rely entirely on diffusion or chaotic advection and do not require external energy except for a micropump for fluid delivery. Despite their higher mixing efficiency, active micromixers and 3D passive micromixers^17^,^18^,^19^ are difficult and costly to be used in LOC applications due to their need for external power sources and/or their sophisticated design and fabrication complexity. Therefore, planar passive micromixers^9^,^10^ are a much more popular choice when applying microfluidics to chemical and biological applications as they are simpler, easy to fabricate and integrable with LOC designs.

The simplest diffusion mixing can be created using two inlet T or Y-shaped microchannels^20^,^21^. Despite its simplicity, more advanced configurations are necessary for an effective diffusion, such as the multi lamination flow, which improves the mixing by increasing the contact surface between two flows^22^,^23^. In this context, hydrodynamic focusing was also utilized as a multi lamination mixing scheme to achieve a complete mixing in the millisecond range^24^,^25^. The chaotic advection is another main approach of mass transfer in the laminar flow and can be obtained by adding obstacle structures inside the channel^26^,^27^,^28^,^29^,^30^. In particular, Hong *et al.*^31^ presented an in-plane modified version of the Tesla structure for flow rates lower than 100 μl/min (Re < 50). In spite of the complexity of the design apt to induce chaotic advection, for Re ≪ 100 the diffusion mixing is still the dominant phenomenon. As another effort, the split and recombination technique was employed to enhance the mixing performance in a planar design with rhombic and circular sub-channels^32^. Among the different solutions, Mengeaud *et al.*^33^ exploited the recirculation phenomenon in a zigzag microchannel, which induces a transversal component of the velocity to improve the mixing process in intermediate-Reynolds-number flows. To sum up, even in the planar configurations the complexity of the device is often adopted to achieve an acceptable mixing level (*i.e.* with mixing index *M* > 90%). Therefore, there is still room to improve the efficiency of the planar passive micromixer in terms of mixing length, mixing time and pressure drop by exploiting an efficient microstructure design. In addition, although the numerical modeling provides valuable predictions for the mixing performance, it is difficult to assess their uncertainty^34^ without considering the experimental challenges (*e.g.* fabrication and characterization), while most of the presented devices just rely on the numerical modeling.

Hereby, we present a novel passive micromixer characterized by a planar effective geometry. This design is a novel layout that combines injection, serial lamination and zigzag-shaped effects to enhance the mixing efficiency. Our work focused on a wide range of numerical modeling and experimental validations, from biochemical mixing to biological applications. We numerically investigated the influence of the geometrical characteristics of the design on the mixing performance over a wide range of flow conditions. The performance of the most efficient design was experimentally investigated by means of fluorescence microscopy for a range of diffusion coefficients, 10^−12^ < *D* < 10^−11^ m^2^/s, and compared with the previous works^17^,^30^,^35^,^36^. Then, we demonstrated the potential diagnostic value of the micromixer by testing its capability for Red Blood Cells (RBC) lysis. The main advantage of the presented design, beside the high mixing efficiency in low-Reynolds-number flows (from *M* = 93% for the mixer including one serpentine to *M* = 99.6% for the mixer including three serpentines), is the easiness of the single-layer-photolithography fabrication process, which paves the way for the integration with LOC devices.

For the design of an efficient passive microfluidic mixer, several aspects, such as mixing science, fluid dynamics and easiness of fabrication procedure for integration with other microfluidic components, should be addressed. We present a 2D laminar micromixer for two contacted fluids, where a solute is concentrated in a solvent with the concentration *c* = *c*_0_ (sample solution) and *c* = 0 (buffer solution), respectively. Figure 1(a) shows the top-view section of the planar microstructure with a rectangular cross-section consisting of a custom zigzag antisymmetric arrangement somewhat similar to an “accordion.”

From the left, the main microchannel is sharply curved and progressively narrowed. From the top, the lateral microchannel connects to the main microchannel in correspondence of the constriction A.

The resulting microchannel is expanded to decrease the flow resistance and then shrunk until the constriction B, where the second lateral microchannel connects in from the bottom (serial lamination technique). The overall arrangement is antisymmetric and, thus, the output microchannel is expanded and curved before the outlet. Both side microchannels are tapered for buffer injection (injection technique). The aim of the combination of constriction A and B is to locally enhance the mean velocity of the fluid and reduce the striation length, which is favorable for the mixing process. In this geometry, the constriction A acts as a Y-junction micromixer, where parallel lamination occurs. Here the sample solution flows with only one diffusion interface (from A to B), the striation length at A is comparable to the half channel width and the constriction B acts as a serial lamination micromixer. It results in two diffusion interfaces (from B on) implying that the striation length is further decreased. Figure 1(b) depicts a more general scheme where a serpentine with *n*_*s*_ repetitions is included in order to lengthen the micromixer and, thus, increase the residence time (*i.e.* the time required for an infinitesimal volume of fluid to reach the outlet in the steady state). Thanks to the sharp bends, the serpentine leads to an enhancement in mixing efficiency for intermediate-Reynolds-number flows (zigzag technique). “*n*_*s*_-SERP” denotes the micromixer type depending on the serpentine repetition unit *n*_*s*_. We considered 0-SERP for numerical analysis and 3-SERP for experimental validation.

The axial length of the device, *L*, and the effective mixing path, *L*_*m*_, are approximately given by


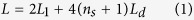



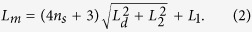


Geometrical parameters are defined in Table 1.

## Results and Discussion

Most passive microfluidic mixers use mainly complex geometry microchannels to increase their mixing performance. Otherwise, mixing occurs predominantly as a result of diffusion effects, hence the mixing channel must have an extended length to achieve a satisfactory performance. Hereby, the mixing performance is enhanced through the microfluidic design by combining injection, serial lamination and zigzag-shaped effects.

**Figure 1:**
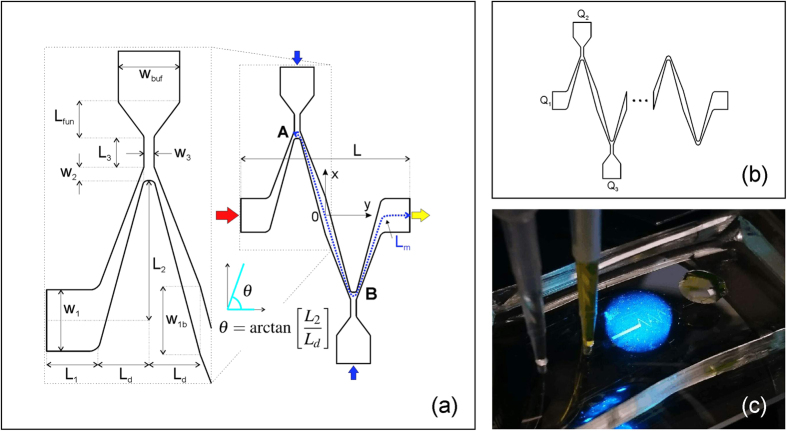
(**a**) Scheme: the sample solution flows in (red arrow); the buffer solution flows in (blue arrows); the mixed solution flows out (yellow arrow); detail of the geometrical parameters (inset); (**b**) Scheme of a serpentine pattern including multiple repetitions (*n*_*s*_-SERP); (**c**) Photographic picture of the fabricated accordion-shaped micromixer (courtesy of A. Cosentino at iit@CRIB, Naples).

A base model of 0-SERP (no repetitions) is defined for simulation (Table 1). The microchannel depth, *H*, is shown only to determine *D*_*h*_ and the dimensionless numbers from Eq. (4). From Eqs (1) and (2), *L* = 1.8 mm and *L*_*m*_ = 2.92 mm and from Eq. (5), *D*_*h*_ ≈ 68.7 *μm*. For sample and buffer solutions, the density is *ρ* = 999.7 kg/m^3^ and the dynamic viscosity is *μ* = 1 × 10^−3^ Pa ⋅ s. Note that we consider a reference value for *D* close to the diffusion coefficient of Bovine Serum Albumin (BSA) protein in water. The effective geometrical parameters (reference values in Table 1)—*e.g.* the accordion angle, *θ* = *arctan*(*L*_2_/*L*_*d*_), and the number of serpentine units, *n*_*s*_, (Fig. 1(a))—affect the mixing performance.

**Figure 2:**
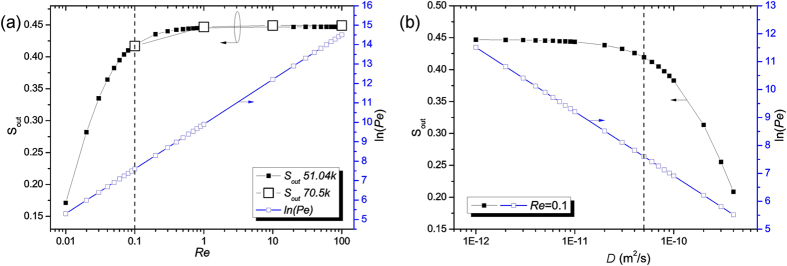
Dependence of the relative variance of the concentration profile at the outlet on Reynolds number and diffusion coefficient. Variation of *S*_out_ on: (**a**) Re (for *n* = 51.04k (solid squares) and *n* = 70.5k (empty squares); reference value: Re = 0.1 (dashed line)) and (**b**) *D* (reference value: *D* = 5 × 10^-11^ m^2^/s (dashed line)). *S*_out_ and ln(Pe) are reported in black on the left y-axis and in blue on the right y-axis, respectively; (0-SERP).

The effect of Péclet number (10^2^ < Pe < 10^6^) and Reynolds number (10^−2^ < Re < 10^2^) variation on micromixer performance are reported in Fig. 2(a,b). The mixing efficiency decreases drastically by up to 30% when increasing Re from 0.01 to 0.1, while this reduction is around 5% in the range of 0.1 to 100. This shows that a high Reynolds number leading to reduction in residence time of the fluid either in diffusion-dominated or advection-dominated mixing process results in an inferior performance. It can be highlighted that with the increment of the Reynolds number, the mixing becomes advection-dominated and the flow is featured by a higher Péclet number. Nonetheless, the nature of the flow is always laminar and only a subtle influence on the concentration profile is found at the constricted and curved regions (patterns not shown). Figure 2(b) shows that with the increment of two orders of magnitude in diffusion coefficient (10^−12^ < *D* < 10^−10^ m^2^/s), the Péclet number decreases considerably to less than 250 and mixing becomes diffusion-dominated with an efficiency higher than 80%. Note that the mixing efficiency increased more than 18% for *D* = 10^−10^ m^2^/s, *i.e.* the most referred diffusion coefficient in the literature. Also, the effect of unbalanced flow rates at the side inlets, *Q*_2_ = *fQ*_1_ and *Q*_3_ = (1 − *f*)*Q*_1_, is analyzed for 0.3 < *f* < 0.7. No remarkable variations in the mixing performance are found.

The performance of the most efficient design was experimentally validated for a wide range of diffusivity, 10^−12^ < *D* < 10^−11^ m^2^/s. Two series of experiments were performed under two different conditions. Two different fluorescent aqueous solutions were prepared by diluting dyed commercial solutes in water: Bovine Serum Albumin (BSA) and 200 nm-diameter Polystyrene (PS) microspheres (see Methods). BSA was selected as a well-known protein standard; PS has the same density as water and is a useful tracer for biomedical purposes. Stokes-Einstein relation was used to estimate the diffusion coefficient of PS aqueous solution. PS microspheres solution was set to a lower concentration to avoid particle aggregation. In any case, although the concentration did not affect the predicted performance, it plays a role in the optical characterization.

**Figure 3:**
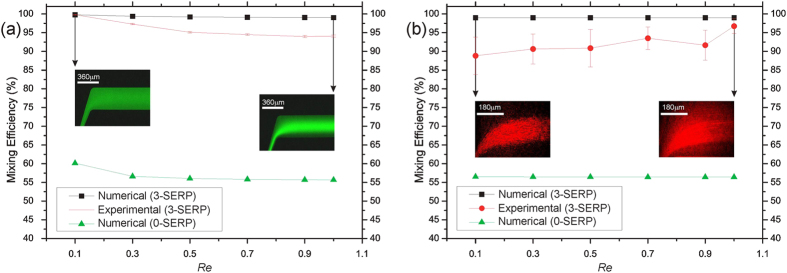
Mixing efficiency for BSA and PS. Variation of *M* as a function of Re for: (a) BSA and (b) PS; Numerical expectations for 0-SERP (green triangles) and 3-SERP (black squares) and experimental dependency for 3-SERP ((a) red error bars; (b) red circles with error bars).

**Figure 4:**
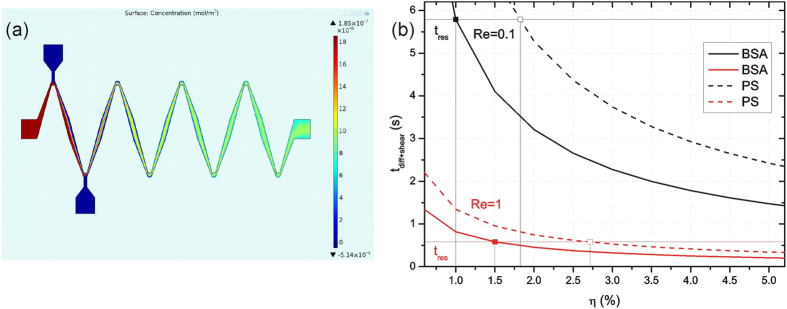
Simulated concentration profile and mixing time. (**a**) Surface plot of the concentration profile *c* simulated for PS (3-SERP); (**b**) Variation of *t*_diff+shear_ as a function of energetic efficiency of mixing, *η*, for Re = 0.1 (black lines) and 1 (red lines) and estimated minimum *η* for BSA (solid squares) and PS (open squares); *t*_res_ = 5.79 and 0.579 s for Re = 0.1 and 1, respectively.

Figure 3(a) compares the numerical results for 0-SERP and 3-SERP and experimental results for 3-SERP related to BSA for Re = 0.1, 0.3, 0.5, 0.7, 0.9, and 1. Analogously, Fig. 3(b) shows the results for PS. Outlet closeups are detailed in the two insets for Re = 0.1 and 1. Differences between experiments and simulations are due to experimental errors. In continuous flow, the optical transfer function of each camera pixel is limited by the exposure time; the correlation between the actual concentration profile and the grayscale profile is no longer valid for low signal-to-noise values. In particular, due to the low concentration of PS microspheres, for low average speed (left inset of Fig. 3(b)) the particles result in a coarse image, while for high average speed (right inset of Fig. 3(b)) the image is smoother. Figure 4(a) shows the surface plot of the numerical concentration profile for PS at Re = 0.1, which qualitatively proves the mixing performance for PS.

The average time that is required for the infinitesimal volume elements of fluid to pass through the device in the steady state is named residence time: *t*_res_ = *L*_*m*_*wH*/*Q*_*t*_ where *Q*_*t*_ is the total flow rate. The expected residence times related to the values of interest of Re and Pe_BSA,PS_ = *Q*_*t*_*D*_*h*_/(*D*_BSA,PS_*Hw*) are reported in Table S2 of Supplementary Information (both for 0- and 3-SERP). Hereby we consider the same assumptions of the theoretical model of Falk and Commenge (see Sec. (2–3)^37^). We impose a laminar shear flow where at least two lamellae are intertwined. The striation length varies in the range from 10 to 30 μm. Moreover, the mixing process is based on the transfer of microfluidic mechanical energy of which only a certain amount is exploited for mixing. Falk and Commenge^37^ defined the energetic efficiency of mixing as (Eq. (9), section 3, page 407) 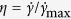, where 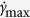 is the total shear rate of the microchannel flow and 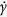 is the shear rate that is indeed used for mixing. According to our numerical predictions, the mean shear rate in the region between the constriction A and the outlet (3-SERP) varies in the range 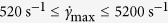 for 0.1 ≤ Re ≤ 1. For Pe_BSA,PS_ ⋅ *η* ≫ 1, the mixing time for the proposed micromixer is^37^


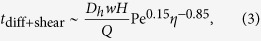


which is a monotonically decreasing function of the energetic efficiency.

Figure 4(b) shows the trends of *t*_diff+shear_ against *η* compared with *t*_res_. The actual value of *η* is not known, but it is fixed for a given Reynolds number. The mixing time for BSA is always lower than the mixing time for PS. Moreover, it is reasonable to state that *t*_diff+shear_ < *t*_res_, otherwise the achieved micromixer performance would not be attained for BSA or PS. A minimum energetic efficiency for efficient mixing can be defined as *η*_min_. According to Fig. 4(b), for Re = 0.1 and 1, *η*_min_ = 1% and 1.5% for BSA and *η*_min_ = 1.8% and 2.7% for PS, respectively. Note that this is only an indicative estimate of *η*_min_ and not the actual energetic efficiency.

Even including a single serpentine unit, the 1-SERP micromixer design is comparable with other planar passive micromixers. For *D* = 5 × 10^−11^ m^2^/s (see Table 1), it reaches the remarkable mixing efficiency of 93% for Re = 0.1 (See section “The role of serpentine repetition units” in Methods), the mixing length of *L*_*m*_ = 6.4 mm (Eq. (2)) and the axial length of only *L* = 3.0 mm (Eq. (1)). This is a considerable improvement compared to the results of former works (11.0 mm^35^ and 32.0 mm^29^), where a higher diffusion coefficient, *D* = 1 × 10^−10^ m^2^/s, was taken into consideration. Clearly, this mixing efficiency and length can be further improved by considering the same diffusion coefficient. Importantly, the use of a simple planar design with comparable length and efficiency to the 3D designs—*e.g.* 5 mm for the staggered herringbone micromixer^36^ and 7 mm for a 3D serpentine laminating micromixer^17^ both offering 90% mixing efficiency—makes it suitable for integration with the other function blocks in LOC devices or batch manufacturing. Furthermore, the potential of the accordion-shaped micromixer as a main block of a diagnostic tool is a plus, which will be demonstrated in the next section.

### Biological application

Micromixers have been growing in several biological applications^38^,^39^,^40^,^41^,^42^. The benefits are low sample/reagent volume, reduced time and risk of contamination, low cost per analysis and portability for point of care (POC) testing. Blood cells counting and purification of nucleic acids from microbial and mammalian cells are crucial steps in many biological, medical^43^,^44^ and forensic^45^ applications. There are different methods to lysate cells^40^. Among them, the chemical approach is the most common procedure thanks to the simplicity, low cost and high percentage of cell lysis. A variety of scientific publications describe microfluidic devices that allow for mixing different solutions^40^. To our knowledge, however, the design and fabrication process of the presented microdevices are quite complex and most of them need to be treated before being functionalized. We investigated the capability of our micromixer for red blood cells (RBC) lysis.

**Figure 5:**
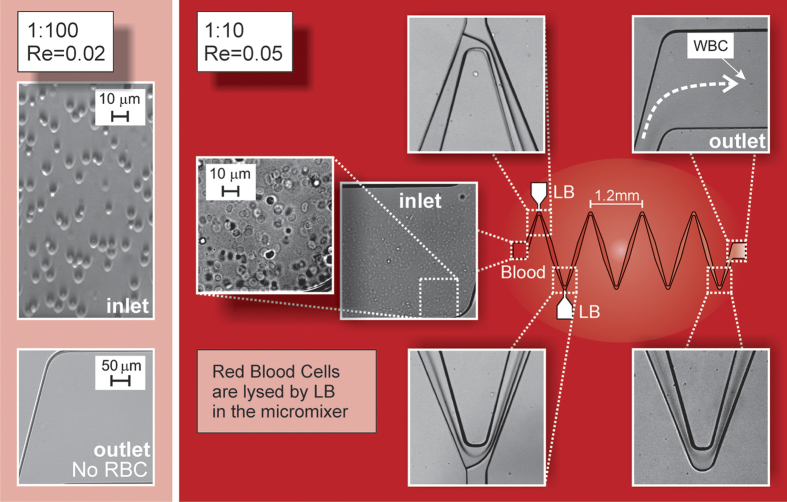
The RBC lysis process in the micromixer. (left-side) Details of RBC concentration at the inlet *vs* lysate sample at the outlet in the case of high-dilution blood sample (1:100); (right-side) Low-dilution blood sample (1:10) with microscopic photographic details: inlet (closeup in the left inset), constriction A and B, last bend and outlet.

For the RBC lysis experiment, we used a specific lysis buffer (see LB in Methods) which does not damage the other blood substances^46^,^47^. The blood sample and lysis buffer were introduced from the main inlet and lateral inlets by means of syringe pumps with a flow rate *Q*_1_ = 0.625 μl/min and *Q*_2_ = *Q*_3_ = 0.3125 μl/min, respectively (Re = 0.05). The imposed flow rate was minimized based on our experimental setup to avoid RBC lysis due to the inertial shear rate. The process of cell lysis was observed and recorded via the presented experimental setup (see Methods). As shown in Fig. 5, during the mixing process the blood sample and LB were efficiently mixed, which led to a complete RBC lysis in the outlet of the microdevice. For the blood sample dilution 1:100 we observed no blood cells in the outlet, while for the lower dilution 1:10 we detected a limited number of cells in the outlet. These might be White Blood Cells, due to the human Red-to-White Blood Cell ratio 1000:1.

The highly efficient RBC lysis experiments is a further confirmation of the capability of the presented micromixer in diagnostic applications. This microdevice is a simple planar and efficient design, which can be integrated easily with other function blocks to create a complete diagnostic system or POC device.

## Conclusions

We designed, fabricated and validated a novel passive planar micromixer. This design is based on several mixing techniques including injection, recombination and zigzag-shaped effects. Moreover, the special microchannel architecture limits the flow resistance thanks to contraction and expansion. The efficient design, based on the mixing performance, is numerically developed in 2D with the study of several geometrical characteristics in the range of 0.01 < Re < 100. The mixing performance of the micromixer is experimentally validated with two well-known standard fluorescent solutes: BSA and PS microspheres in a range of low diffusion coefficients 10^−12^ < *D* < 10^−11^ m^2^/s. Our experimental results show a very good agreement with the simulations: the difference is always lower than 10%. One can take advantage of the accordion-shaped design by choosing the number of serpentine units based on a compromise between the required mixing efficiency and the pressure drop. For instance, the micromixer including a single serpentine unit enables to achieve an efficiency of 90% with the acceptable pressure drop of 500 Pa at Re = 0.1 and easily reaches almost 100% increasing the serpentine repetition unit. Finally, the high mixing efficiency of the presented microchip paves the way for the investigation of RBC lysis as a crucial step for extraction and analysis of the intercellular components. The complete RBC lysis process is a strong evidence of the potential diagnostic value and capability of the micromixer for POC testing.

## Methods

### Dimensional numbers and hydraulic diameter

The characteristic dimensionless numbers are the Reynolds number, the Péclet number and the Schmidt number


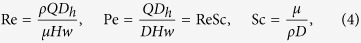


where *H* is the depth, *w* is the width, *Q* is the flow rate, *ρ* is the density, *μ* is the dynamic viscosity, *D*_*h*_ is the hydraulic diameter and *D* is the diffusion coefficient of the solute in the solvent. Note that Sc depends only on the characteristics of the sample and buffer solutions.

In this article, Re and Pe are defined at the main inlet with *w* = *w*_1_ and *Q* = *Q*_1_. The numerical simulations are performed in a 2D model and the channel depth, *H*, is introduced exclusively to determine Re, Pe (Eq. (4)) and *D*_*h*_ (Eq. (5)). For a rectangular-shaped microchannel,


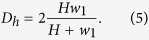


### Numerical model

The Finite Element Method (FEM) is used with a numerical model of laminar mixing of incompressible fluids. The fluidic transport process is simulated to determine the velocity field, the pressure and the concentration. The numerical simulations are carried out by using the numerical code from the Computational Fluid Dynamics (CFD) module of a commercial software (COMSOL Multiphysics 4.4^48^). First, the solutions for the continuity equation and the steady Navier-Stokes equation are found (incompressible laminar flow). Second, the concentration profile, *c*, is determined by solving the stationary diffusion-convection equation. The boundary conditions are imposed: (i) the inflow is laminar for each inlet (we define *Q*_1,2,3_ (Fig. 1b) and impose the no-slip condition at the sidewalls) and (ii) *p* = 0 at the outlet. Moreover, it is assumed that: (i) variations of concentration (for *c*_0_ ≤ 5 × 10^−2^ mol/m^3^) do not affect *μ* and *ρ* (transport of diluted species); (ii) the sidewalls are smooth; and (iii) the surface tension forces of the walls as well as the body forces are neglected.

The mixing efficiency is defined as


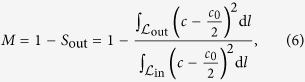


where *S*_out_ is the relative variance of the concentration profile at the outlet, 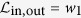 are the total inlet and outlet length (perpendicular section), respectively. The average concentration after perfect mixing is *c*_0_/2.

### The mesh

We defined a 2D discretization mesh, where extremely high refinement is required only along the diffusion interfaces (the resolution requirements of the Navier-Stokes equation are less restrictive than those of the convection-diffusion equation). Free quad meshes are generated with different resolutions in separated subdomain portions. For large total number of mesh elements *n* ≥ 51.04 *k*: (i) *S*_out_ is constant (*S*_out_ = 0.419) (see Fig. S1 in Supplementary Information) and (ii) *c* at the outlet line exhibits negligible deviation. Figure 2(a) compares numerical data for *n* = 51.04 *k* and *n* = 70.5 *k* for Re = 0.1, 1, 10, and 100. The mesh with *n* = 51.04 *k* elements is chosen for quick calculations.

### The role of accordion angle (*θ*)

**Figure 6:**
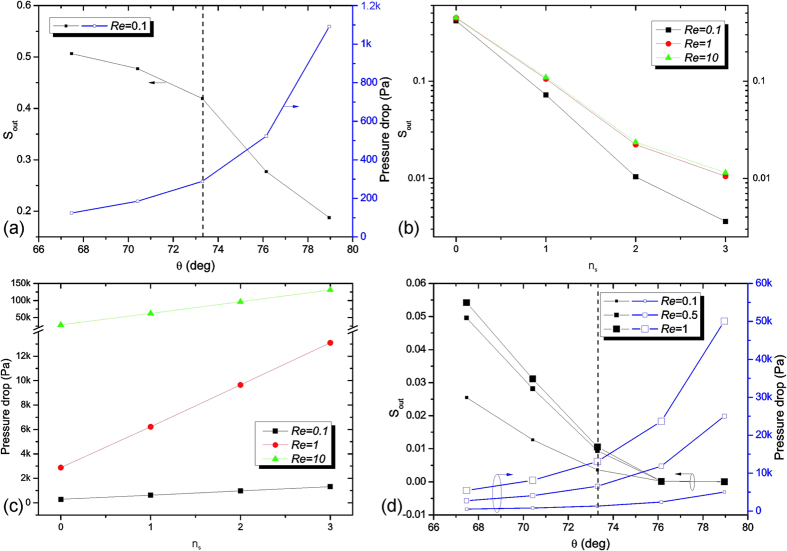
Dependence of *S*_out_ and pressure drop on accordion angle and serpentine repetition units. (**a**) *S*_out_ (in black on left y-axis) and Δ*p* (in blue on right y-axis) as a function of angle *θ* (67.5°, 70.4°, 73.3° (dashed line), 76.1° and 79.0°) for 0-SERP; (**b**) *S*_out_ and (**c**) Δ*p* as a function of *n*_*s*_ (0, 1, 2, 3) for *n*_*s*_-SERP for Re = 0.1, 1, and 10; (**d**) *S*_out_ (in black on left y-axis) and Δ*p* (in blue on right y-axis) as a function of *θ* for 3-SERP for Re = 0.1, 0.5, and 1.

The accordion angle is varied by keeping *L*_*m*_ = 2.92 mm constant (Eq. (2)). Figure 6(a) shows *S*_out_ and Δ*p* as a function of *θ* for Re = 0.1. For large angles, the performance is higher at the expense of the pressure drop (vice versa for small angles). *S*_out_ can be improved by up to 60% by increasing *θ* from 67° to 79° at the cost of Δ*p* up to 1 kPa. A trade-off is found between high mixing efficiency and low pressure drop. We refer to θ^*^ = 73.3°, which improves the mixing efficiency by almost 10% increasing the pressure drop no more than 200 Pa. If *θ* > θ^*^, Δ*p* is drastically increased.

### The role of serpentine repetition units (*n*_*s*_)

Figure 6(b) demonstrates that the mixing efficiency increases with the number of serpentine repetition units, *n*_*s*_. Also Δ*p* increases with *n*_*s*_ (Fig. 6(c)). Passing from *n*_*s*_ = 0 to 1, a mixing efficiency higher than 90% is promptly achieved. Even for *n*_*s*_ = 3 and Re = 10, Δ*p* does not exceed more than 150 kPa. Figure 6(d) shows the performance of 3-SERP (*L*_*m*_ = 13.4 mm) corresponding to the variation of *θ* for *D* = 5 × 10^−11^ m^2^/s and Re = 0.1, 0.5, and 1. The obtained results indicate that passing from *θ* = 67° to *θ* = 79°, the mixing efficiency is always higher than 94%, while the pressure drop is always lower than 50 kPa for Re = 0.1, 0.5, and 1.

### Fabrication methods

The micromixer chip consists of a slab of poly-dimethylsiloxane (PDMS) bonded onto glass. A 50 μm-thick hollow microchannel is patterned into the slab and capped with the glass substrate. The fabrication process is based on maskless photolithography and soft lithography. A master consisting of photoresist protrusions over a silicon substrate is fabricated for PDMS replication. The master is micropatterned by means of the Direct Writing Laser system (Heidelberg DWL66FS, www.himt.de). The negative tone photoresist mr-DWL-40 (from www.microresist.de) is spun onto a 2-inch Si wafer and soft-baked on hotplate at 50 °C – 85 °C thermal ramp for 15 min. Light exposure occurs at the optical wavelength of ~405 nm. Then, the mold is post-baked at 50 °C −85 °C thermal ramp for 15 min and developed in mr-Dev-600 (www.microresist.de) soak bath for 2 min to release the unexposed material. The PDMS (Sylgard 184, Dow Corning) elastomer base is first mixed with the curing agent in a ratio 10:1. Afterwards, the mixture is centrifuged at 1200 rpm for 4 min for degassing and then poured over the mold. PDMS cross-linking occurs in oven at 90 °C for 60 min. The structured PDMS slab is gently demolded and inlets and outlet are punched by means of a 1 mm-diameter needle. The glass slide and the PDMS slab are treated with O_2_ plasma process for surface activation. Glass-to-PDMS bonding occurs as soon as the interfaces are in contact with each other. Finally, the device is put in oven at 85 °C for 15 min and complete bonding is achieved. Figure 1(c) shows a photographic picture of the fabricated device.

### Experimental setup

The experimental setup included a syringe pump apparatus (NEMESYS, Germany NEMESYS syringe pumps systems), a fluorescence direct inverted microscope (Olympus, Japan www.olympus.com), a high-speed CCD camera (Imperx Inc., USA) and a computer.

### Microfluidic setup and optical characterization

BSA and PS solutions were mixed with water (see Table S1 in Supplementary Information). BSA and PS were conjugated with different fluorochromes: FITC (albumin-fluorescein isothiocyanate conjugate – SIGMA-ALDRICH) and Rhodamine B (Firefli Fluorescent Red – Duke Scientific Corporation), respectively. We employed three external syringe pumps to control the flow rates at the three inlets: *Q*_1,2,3_. If *Q*_1_ = *Q*_2_ + *Q*_3_, equal volumes of sample and buffer solution flowed through the outlet per unit time. Moreover, we set *Q*_1_ = 2*Q*_2_ = 2*Q*_3_. A glass syringe was filled with the BSA (or PS) solution and two glass syringes were filled with water. First, the whole device was filled only with water in order to void air pockets. Plastic tubes were used as port connections to deliver liquids to the inlets. The outlet was left with no connection to impose the atmospheric pressure. Second, *Q*_1,2,3_ were set and the steady state was reached after five minutes. *Q*_1_ = 1.25 μl/min corresponded to Re = 0.1. A FITC (TRITC) filter set for BSA (PS) was mounted in the microscope. The fluorescent signal was captured by the camera from the top of the device. Images were analyzed to determine the experimental concentration profile.

### Signal processing

We used “ImageJ” software for image processing. The concentration of BSA (PS) was directly determined from the post-processed optical signal. Negligible variations in the mixing quality along the channel depth were assumed. More generally, the signal might depend on the illumination condition, the actual angle of view of the microscope, and the focal depth. We considered transverse lines at the inlets/outlet channels. For the two experiments, the microchannel width of 360 μm covered 190 pixels (insets of Fig. 3(a)) and 467 pixels (insets of Fig. 3(b)), respectively. The grayscale values at the outlet (*g*_out_) were normalized with respect to the grayscale values at the inlets (*g*_in1_ for sample inlet and *g*_in2_ for water inlet).

The normalized concentration *c*_*N*_ at the transverse outlet line is


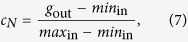


where *min*_in_ and *max*_in_ are the minimum and maximum of *g*_in1_ and *g*_in2_, respectively. The mixing quality *M* is *M* = 1 − *S*_out_, where *S*_out_ (Eq. (6)) is calculated as the variance of *c*_*N*_, *S*_out_ = Var(*c*_*N*_). Error bars in Fig. 3 are the standard deviations over M obtained for more than 30 line profiles.

### Blood experiment

Fresh human blood was collected from a healthy volunteer via finger prick method using a lancelet of a POC device. Then, two types of blood samples were prepared by mixing with Heparin (purchased from Sigma Aldrich) as standard anticoagulant in 1:10 and 1:100 dilution ratio. The LB for RBC was prepared by mixing: 320 mM Sucrose, 5 mM MgCl_2_, 10% TritonX-100 and 10 mM Tris HCl pH 7.8. The advantage of this buffer is that there is no damage to the other blood substances^46^,^47^.

## Additional Information

**How to cite this article**: Cosentino, A. *et al.* An efficient planar accordion-shaped micromixer: from biochemical mixing to biological application. *Sci. Rep.* **5**, 17876; doi: 10.1038/srep17876 (2015).

## Supplementary Material

Supplementary Information

## Figures and Tables

**Table 1:** Geometric and reference parameters of 2D base model.

Parameter	Description	Value
*w*_1_	Main inlet width	360 μm
*w*_buf_	Buffer inlet width	360 μm
*w*_2_	Main constriction width	80 μm
*w*_3_	Buffer constriction width	60 μm
*w*_1*b*_	Max decompression width	400 μm
*L*_1_	Main inlet length	300 μm
*L*_fun_	Funnel length	200 μm
*L*_2_	Lateral shift length	820 μm
*L*_3_	Buffer constriction length	180 μm
*L*_*d*_	Longitudinal shift length	300 μm
*H*	Microchannel depth	38 μm
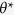	Angle (*arctan*(*L*_2_/*L*_*d*_))	73.3°
Re	Reynolds number	0.1
*D*	Diffusion coefficient	5 × 10^−11^ m^2^/s
*c*_0_	Initial concentration	0.01 mol/m^3^
*n*	Mesh elements	≈51041
