# Supplementary material for: An efficient planar accordion-shaped micromixer: from biochemical mixing to biological application
Source: Sci Rep. 2015 Dec 14;5:17876. doi: 10.1038/srep17876 (PMC4677335; doi:10.1038/srep17876)
Supplement: Supplementary Information [file srep17876-s1.pdf]

SUPPLEMENTARY INFORMATION to: An efficient planar accordion-shaped micromixer: from biochemical mixing to biological application

October 19, 2015

Armando Cosentino,[a12] **Hojjat Madadi,**[a123] **Paola Vergara,**[a] **Raffaele Vecchione,**[a] **Filippo Causa,**[a,b,c] **and Paolo A. Netti**[a,b,c2]
[a] *Center for Advanced Biomaterials for Healthcare@CRIB, Istituto Italiano di Tecnologia (IIT), Largo Barsanti e Matteucci 53, 80125 Naples, Italy.*
[b] *Interdisciplinary Research Centre on Biomaterials (CRIB), University "Federico II", Piazzale Tecchio 80, 80125 Naples, Italy*
[c] *Dipartimento di Ingegneria Chimica, dei Materiali e della Produzione Industriale (DICMAPI), University "Federico II", Piazzale Tecchio 80, 80125 Naples, Italy*

Table S 1: Aqueous liquid samples for experimental validation: initial concentration, diffusion coefficient and Schmidt number (= Pe/Re) at 20°.

Solute	c_0 (mol/m^3)	D (m^2/s)	Sc (−)
BSA	3.8×10^{-2}	6×10^{-11}	1.67×10^{4}
PS	1.8×10^{-7}	2.2×10^{-12}	4.59×10^{5}

[1] *Both authors Armando Cosentino and Hojjat Madadi have equally contributed to this work.*

[2] armando.cosentino@iit.it (A.Cosentino), hojjat.madadi@espci.fr (H.Madadi), antonio.netti@iit.it (P.A.Netti)

[3] Laboratoire de Colloïdes et Matériaux Divisés (LCMD), Ecole Supérieure de Physique et Chimie Industrielles de la ville de Paris (ESPCI), Paris, France.

Table S 2: Estimated residence time for different values of Re and Pe and micromixer design types.

Reynolds number Re	Total flow rate Q_t (μl/min)	Péclet number BSA $\mathrm{Pe}_{\mathrm{BSA}}$	Péclet number PS $\mathrm{Pe}_{\mathrm{PS}}$	Residence time 0-SERP $t_{\mathrm{res},0}$ (ms)	Residence time 3-SERP $t_{\mathrm{res},3}$ (ms)
0.1	2.5	1670	45900	1262	5789
0.3	7.5	5010	137700	421	1930
0.5	12.5	8350	229500	252	1158
0.7	17.5	11690	321300	180	827
0.9	22.5	15030	413100	140	643
1	25	16700	459000	126	579

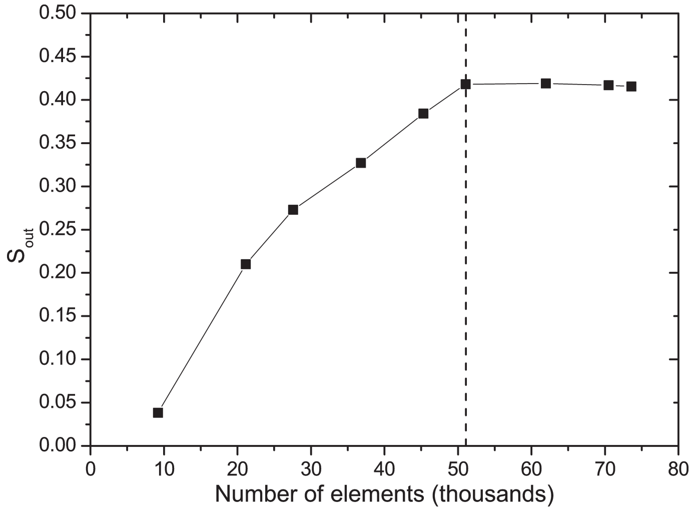

Fig. S 1: Variation of S_{out} as a function of number of mesh elements; Reference value: $n = 51.04k$ (dashed line); (0-SERP).
